# Coverage and efficacy of intermittent preventive treatment with sulphadoxine pyrimethamine against malaria in pregnancy in Côte d’Ivoire five years after its implementation

**DOI:** 10.1186/s13071-014-0495-5

**Published:** 2014-11-20

**Authors:** Offianan A Toure, Penali L Kone, M’Lanhoro AA Coulibaly, Berenger AA Ako, Eric A Gbessi, Baba Coulibaly, Landry T N’ Guessan, David Koffi, Sylvain Beourou, Adama Soumahoro, Issiaka Bassinka, Messoun Nogbou, Tidjane Swa, Bernadin Gba, Beugre Esmel, Ernestine M Bokossa

**Affiliations:** Malariology Department, Institut Pasteur of Côte d’Ivoire, P.O. Box 490, Abidjan, 01 Côte d’Ivoire; PMI of Yopougon Abidjan, P.O. Box 1351, Abidjan, 23 Côte d’Ivoire; Maternity Service, Bonoua Hospital, P.O. Box 25, Bonoua, Côte d’Ivoire; CHU Cocody, Boulevard de l’Université, P.O. Box v13, Abidjan, Côte d’Ivoire

**Keywords:** Coverage, IPTp-sp, Malaria, Efficacy, Côte d’Ivoire

## Abstract

**Background:**

The World Health Organization (WHO) recommends for sub-Saharan Africa a package of prompt and effective case-management combined with the delivery of insecticide-treated nets (ITN) and intermittent preventive treatment during pregnancy (IPTp) with sulphadoxine-pyrimethamine (SP) through the national antenatal care (ANC) programs. Implemented in Côte d’Ivoire around 2005, few Data on IPTp coverage and efficacy in the country are available.

**Methods:**

A multicentre, cross-sectional survey was conducted in Côte d’Ivoire from September 2009 to May 2010 at six urban and rural antenatal clinics. IPTp-sp coverage, Socio-economic and obstetrical data of mothers and neonate birth weights were documented. Peripheral blood as well as placental and cord blood were used to prepare thick and thin blood films. In addition, pieces of placental tissues were used to prepare impression smears and maternal haemoglobin concentration was measured. Regression logistics were used to study factors associated with placental malaria and LBW (<2.500 grams).

**Results:**

A total of 1317 delivered women were enrolled with a median age of 26 years. A proportion of 43.28% of the women had received at least two doses of IPTsp during the current pregnancy although a high proportion (90.4%) of women received antenatal care and made enough visits (**≥**2). Variability in the results was observed depending on the type of area (rural/urban). *Plasmodium falciparum* was detected in the peripheral blood of 97 women (7.3%) and in the placenta of 119 women (9%). LBW infants were born to 18.8% (22/107) of women with placental malaria and 8.5% (103/1097) of women without placental malaria. LBW was associated with placental malaria.

**Conclusions:**

This study found relative low coverage of IPTp in the study areas which supported findings that high ANC attendance does not guarantee high IPTp coverage. Urgent efforts are required to improve service delivery of this important intervention.

## Background

Malaria is one of the most important preventable causes of poor maternal health and adverse birth outcomes [1]. In sub-Saharan Africa, an estimated 30 million pregnant women are in danger of *Plasmodium falciparum* infections every year [2].

To prevent malaria during pregnancy and its adverse outcomes, the World Health Organization (WHO) recommends the use of intermittent preventive treatment with sulfadoxine-pyrimethamine (IPTp-SP), sleeping under an insecticide treated bed net (ITN) and prompt management of malaria cases and anaemia [3].

ITPp consists of administering, to all pregnant women, at least two doses of intermittent preventive treatment with sulfadoxine-pyrimethamine (IPTp/SP) during the second and third trimesters of pregnancy during routinely scheduled antenatal clinic visits irrespective of the presence of signs for a malaria infection [4,5].

Clinical trials and program evaluations in stable transmission areas have shown that this intervention is safe, efficacious, and effective in preventing maternal anemia, placental parasitemia, and LBW [6-8].

Côte d’Ivoire has implemented the WHO recommendations for preventing malaria since 2005. Few data regarding IPTp-SP coverage and its efficacy are available since its implementation in the country [9,10].

It therefore seems important to assess the ITP situation in the country fives years after the adoption of this policy by the National Malaria Control Program.

The objective of this study was to evaluate the coverage of IPT-SP and its efficacy five years after its implementation in Côte d’Ivoire.

## Methods

### Study sites

The study was carried out in urban (PMI of Yopougon, Fsucom of Anonkoua-kouté, CHU Cococdy, FSUCOM Abobo PK18) and rural areas (Hospital of Bonoua, CSU of Yaou CSU of Samo). In the study sites, perennial malaria transmission with seasonal peaks is mostly attributable to *P. falciparum*.

### Study population

Pregnant women who gave birth at any of the six study clinics during the study period, gave their written informed consent, and donated placentas for blood collection were enrolled.

### Sample size calculation

The sample size calculation was based on the estimate of a proportion of placental malaria. The prevalence of placental malaria after IPT-SP implementation was approximatively 10% [9]. With a margin of error of ±2% using an alpha type-1 error of 5%, at least 1120 delivered pregnant women should be included.

### Delivery

During labor, venous blood samples were collected for determination of Hb concentration and malaria diagnosis. Immediately after delivery, babies were weighed using a hanging weighing scale (Model 180; Salter Brecknell, West Midlands, United Kingdom). Data regarding newborn characteristics (vital status at birth, birth weight, sex, and the presence of twins or malformation) were collected. Blood smears were made with blood collected from the maternal side of the delivered placenta and the umbilical vein cord. In addition, pieces of placental tissues were used to prepare impression smears after swabbing it on blotting paper.

### Laboratory procedures

Thick films and placental impression smears were stained with 10% Giemsa for 15 minutes. To determine the percentage of malaria parasitemia from placental impression smears, malaria parasite-infected red cells were counted against 2000 erythrocytes.

Placental infection status was categorized as infected (presence of any asexual parasite stages in the placenta) and noninfected (parasite negative smear). Smears with malaria pigment but with no asexual parasite stages were declared as unknown and were not included in the analysis. Malaria pigment is a sign of malaria infection during pregnancy. We would consider these samples as positive.

Microscopic examination of blood smears was done under oil immersion for parasite detection and 200 high-power fields were examined before the smear was considered negative.

Parasites were enumerated using thick film, as previously described [11]. The parasite density (per 1 μL of blood) was calculated, assuming a normal leukocyte level of 8000/μL.

The thin film was used to speciate the parasites. Each blood film was independently examined by two microscopists. In cases of discrepancy, a third microscopist counted the number of parasites in the films. The average of two counts that agreed was used as the final level of parasitemia. Venous blood (5 mL) was collected using butterfly needles into ethylenediaminetetraacetic acid Vacutainer® tubes (BD Diagnostics, Franklin Lakes, NJ) for measurement of Hb levels using an automated hematology analyzer (Mythic 22; Orphee SA, Geneva, Switzerland).

### Ethical clearance

The study was approved by the Comité National d’Ethique et de Recherche (CNER) of Côte d’Ivoire. All study participants were informed in their local language about the study objectives and procedures. For each study participant, written informed consent was obtained and the participant was free to withdraw consent at any time of the study without influencing their access to health services.

### Statistical analysis

We classified participants as primigravidae (first pregnancy), secondigravidea (second pregnancy) and multigravidae (third and subsequent pregnancy). Doses of SP IPTp were classified as no SP treatment, 1 dose, 2 doses, or **≥**3 doses. Bed net usage was coded as whether or not the woman usually slept under a bed net. We defined anemia as hemoglobin concentration, 11 g/dl and low birth weight (LBW) as infant’s birth weight <2500 g.

The exposure variables of interest were SP IPTp (0, 1, 2 or **≥**3 doses) and bed net use. Outcome variables were peripheral parasitemia (detection of parasites in peripheral blood), placental parasitemia (detection of parasites in placental blood), maternal hemoglobin concentration at delivery and anemia, infant’s birth weight and LBW.

Differences in frequencies were compared by either chi-squared or Fisher’s exact tests as appropriate, and continuous variables by Student’s *t*-test when the data were normally distributed. Nonparametric tests were used for non normally distributed data. In the multivariable analysis, the factors associated with the dependant variable (LBW or placental malaria) based on univariable analysis were included. Statistical analysis was performed using Stata® version 10.0 (StataCorp LP, College Station, TX).

## Results

### Characteristics of the study participants at enrollment

A total of 1317 (815 from urban area and 502 from rural area) delivered women were enrolled. Characteristics of the mothers are summarized in Table 1. The mean age of the participants was 26 years. Primigravidae and secundigravidae represent respectively 24.1% and 25.1% of the total sample size.

**Table 1:** **Socio-demographic characteristics in delivered women according to the number IPT-SP doses (N =1317)**

		**IPTp-sp dose**				
	**Total**	**0**	**1**	**2**	**≥3**	***P-value***
	**N = 1317**	**n = 437**	**n = 310**	**n = 483**	**n = 87**	
**Age (year)**						
Médian (±SD)	26 (6.12)	25 (6.14)	26 (6.42)	26 (5.85)	27 (6.15)	0.01
<20	160 (12.1)	61 (13.9)	42 (13.5)	42 (8.6)	15 (17.2)	0.02
20-24	401 (30.4)	151 (34.5)	89 (28.7)	146 (30.2)	15 (17.2)	0.01
25-29	346 (26.2)	104 (23.7)	76 (24.5)	139 (28.7)	27 (31.0)	0.21
≥30	410 (31.1)	121 (27.6)	103 (33.2)	156 (32.2)	30 (34.4)	0.28
**Gravitidy**						
Médiane (±SD)	3 (1.90)	3 (1.98)	3 (2.01)	3 (1.75)	2 (1.68)	0.11
1	317 (24.0)	104 (23.7)	69 (22.2)	115 (23.8)	29 (33.3)	0.19
2	331 (25.1)	105 (24.0)	76 (24.5)	125 (25.8)	25 (28.7)	0.78
≥3	669 (50.7)	228 (52.1)	165 (53.2)	243 (50.3)	33 (37.9)	0.07
**Number of ANC visit**						
Médian (±SD)	4 (1.66)	4 (1.63)	3 (1.74)	4 (1.65)	5 (1.62)	<0.0001
0	17 (1.4)	17 (3.8)	00	00	00	<0.0001
1-3	603 (45.7)	198 (45.3)	189 (60.9)	153 (31.6)	63 (72.4)	<0.0001
≥4	697(52.9)	222 (50.8)	121 (39.0)	330 (68.3)	24 (27.5)	<0.0001
**Birth weight (grams)**						
Médiane (±SD)	3000 (453.04)	3000 (487.03)	3100 (419.09)	3000 (374.30)	3150 (274.93)	<0.0001
<2500	111 (8.46)	50 (11.46)	19 (6.20)	39 (8.07)	3 (3.44)	0.009
≥2500	1201 (91.54)	386 (88.54)	287 (93.80)	444 (91.93)	84 (96.56)	-
**Locality**						
ANK	105 (7.9)	35 (8.0)	26 (8.3)	39 (8.0)	5 (5.7)	0.88
BONOUA	257 (19.5)	23 (5.2)	99 (31.9)	124 (25.6)	11 (12.6)	<0.0001
COCODY	195 (14.8)	32 (7.3)	54 (17.4)	109 (22.5)	0 (00)	<0.0001
SAMO	81 (6.1)	05 (1.1)	28 (9.0)	31 (6.4)	17 (19.5)	<0.0001
YAOU	164 (12.4)	08 (1.8)	25 (8.0)	85 (17.5)	46 (52.8)	<0.0001
YOPOUGON	515 (39.1)	334 (76.4)	78 (25.1)	95 (19.6)	8 (9.1)	<0.0001

### Number of ANC visits

A total of 1300 women attended antenatal clinics at least once during pregnancy but only 75.3% completed the recommended three or more ANC visits.

A high proportion (90.4%) of women received antenatal care and made enough visits (**≥**2).

### Coverage of ITP-sp

A total of 880 among 1317 delivered women (66.8%) received ≥1 dose of IPT-SP. The IPT-SP coverage (≥2 doses) was 43.3% (570/1317) and varied according to the study site, the number of ANC visits and age of mother (Table 1).

IPTp coverage rate (≥2 doses) was higher in rural areas (47.8%) than in urban areas (29.8%) (p <0.0001). However, the highest rate was recorded at Cocody site (55.9%), followed by Yaou (51.8%), and Bonoua (48.2) and the lowest rate was observed in Abobo PK18 (14.3) and Yopougon (18.5%).

Although 98.7% (1300/1317) of recently pregnant women visited ANC at least once in an eligible trimester, only 67.7% received the first dose of SP, and of the 91.5% (1190/1300) of women who returned for a second ANC visit, only 48.0% (571/1190) received a second dose.

### Low birth weight

LBW infants were born to 19.7% (23/117) of women with placental malaria and 8.6% (103/1200) of women without placental malaria (p < 0.0001).

The factors associated with LBW in the multivariable analysis were placental malaria ( adjusted odds ratio: 2.7; 95% confidence interval: [1.6-4.6], maternal malaria during pregnancy (adjusted odds ratio: 4.0; 95% confidence interval: [1.4–4.2], untake IPTp-SP (adjusted odds ratio: 5.8; 95% confidence interval: [1.4–24.6], Use IPT-sp (adjusted odds ratio: 4.8; 95% confidence interval: [1.1-20.5] and maternal malaria at delivery (adjusted odds ratio: 2.4; 95% confidence interval: [1.4–4.2] (Table 2).

**Table 2:** **Factors associated with LBW: logistic regression model (N =1 312)**

		**Weight <2500 g**		**Univariate analysis**				**Multivariate analysis**			
**Factors associated**	**N**	**n**	**%**	**OR**	**95% IC**		***P-value***	**AOR**	**95% IC**		**P-value**
**Parity**											
Primiparous	306	38	12.42	2.12	1.32	4.42	*0.0019*	2.03	1.25	3.31	*0.0042*
Secondiparous	414	36	8.70	1.42	0.88	2.30	*0.14*	1.32	0.85	2.14	*0.25*
Multiparous	592	37	6.25	-	-	-	*-*	-	-	-	*-*
**IPTp-sp**											
0	436	50	11.46	-	-	-	*-*	-	-	-	*-*
1	306	19	6.20	0.52	0.30	0.90	*0.02*	0.54	0.31	0.96	*0.03*
2	483	39	8.07	0.69	0.44	1.07	*0.10*	0.70	0.44	1.12	*0.14*
≥3	87	03	3.44	0.27	0.08	0.91	*0.03*	0.24	0.07	0.81	*0.02*
**Number of ANC visit**											
0	16	3	18.75	-	-	-	*-*	-	-	-	*-*
1	119	10	8.40	0.53	0.13	2.16	*0.38*	-	-	-	*-*
2	197	15	7.61	0.46	0.12	1.77	*0.26*	-	-	-	*-*
≥3	980	89	9.08	0.52	0.15	1.82	*0.31*	-	-	-	*-*
**Placental malaria**											
Positive	117	22	18.80	2.87	1.72	4.79	*0.0001*	2.29	0.81	6.45	*0.11*
Negative	1195	89	7.45	-	-	-	*-*	-			*-*
**Malaria during pregnancy**											
Positive	154	18	11.68	1.47	0.86	2.52	*0.15*	1.19	0.68	2.10	*0.53*
Négative	1158	93	8.03	-	-	-	*-*	-			
**Peripheral malaria**											
Positive	97	18	18.55	3.08	1.76	5.38	*0.0001*	1.30	0.42	4.06	*0.64*
Negative	1215	93	7.65	-	-	-	*-*	-			
**Anemia at delivery**											
Severe anemia	203	23	11.33	1.35	0.77	2.37	*0.29*	-	-	-	*-*
Moderate anemia	552	47	8.51	0.94	0.59	1.51	*0.82*	-	-	-	*-*

### Placental malaria

*Plasmodium falciparum* was detected in the peripheral blood of 97 women (7.3%) and in the placenta of 119 women (9.0%). Among the 97 mothers with peripheral malaria parasitaemia, 29 (6.6%) did not receive IPT-SP and 45 (7.9%) received at least two doses of SP (p = 0.45). Placental impression smears were positive for 56 (9.8%) women who had received at least two doses of SP and for 36 (8.2%) women who had not received IPT-SP (p = 0.39).

Prevalence of placental malaria was higher in rural areas (11.8% *vs* 7.1%; p =0.004). The prevalence of women with peripheral parasitemia was 10.8% (33/303), 6.9% (29/420), and 5.8% (35/594) respectively in primigravidae, secondigravidae, and multigravidae (p = 0.04). Slightly higher prevalence of placental malaria was recorded with 11.8% (35/303), 9.7% (41/420), and 7.2% (43/594) respectively in primigravidae, secondigravidae and multigravidae women (p = 0.08). The factors associated with placental malaria in multivariable analysis, were malaria during pregnancy (adjusted odds ratio: 2.0; IC 95%: [1.2-3.5], moderate anemia (Hb < 11 g/dL)(adjusted odds ratio: 0.4; Interval Confidence 95%: [0.2-0.7], gravidy (adjusted odds ratio: 1.7; IC 95%: [1.1-2.6], Yopougon locality (adjusted odds ratio: 0.5; IC 95%: [0.3-0.9] (Table 3).

**Table 3:** **Factors associated with placental malaria: logistic regression model (N =1 317)**

		**Placental malaria**		**Univariate analysis**				**Multivariate analysis**			
**Factors associated**	**N**	**n**	**%**	**OR**	**95% IC**		***P-value***	**AOR**	**95% IC**		***P-value***
**Age (year)**											
<20	160	18	11.25	1	-	-	*-*	1	-	-	*-*
20-24	401	50	12.46	0.98	0.55	1.73	*0.94*	1.06	0.60	1.89	*0.82*
25-29	346	18	5.20	0.49	0.25	0.93	*0.03*	0.52	0.27	1.00	*0.05*
≥30	410	31	7.56	0.59	0.32	1.09	*0.09*	0.64	0.34	1.20	*0.16*
**Gravitidy**											
1	317	38	11.98	1.2	0.74	1.93	*0.44*	-	-	-	*-*
2	331	30	9.06	1.4	0.91	2.23	*0.11*	-	-	-	*-*
≥3	669	49	7.32	1	-	-	*-*	-	-	-	*-*
**Locality**											
ANK	105	8	7.62	1	-	-	*-*	-	-	-	*-*
Bonoua	257	28	10.89	1.46	0.67	3.19	*0.33*	1.46	0.67	3.21	*0.33*
Cocody	195	16	8.20	0.82	0.34	1.97	*0.66*	0.91	0.37	2.20	*0.83*
Samo	81	6	7.40	1.85	0.74	4.64	*0.18*	1.83	0.72	4.62	*0.19*
Yaou	164	25	15.24	1.48	0.64	3.39	*0.35*	1.54	0.67	3.54	*0.30*
Yopougon	515	34	6.60	0.68	0.31	1.48	*0.33*	0.68	0.31	1.49	*0.34*
**IPTp-sp**											
0	437	34	7.78	1	-	-	*-*	-	-	-	*-*
1	310	26	11.92	1.04	0.61	1.75	*0.87*	-	-	-	*-*
≥2	570	57	10.00	1.19	0.77	1.85	*0.41*	-	-	-	*-*
**Malaria during pregnancy**											
Positif	154	11	7.14	0.66	0.34	1.30	*0.24*	-	-	-	*-*
Négatif	1163	106	9.11	1	-	-	*-*	-	-	-	*-*
**Anemia at delivery**											
Severe anemia <8 g/dL	203	35	17.24	4.86	2.67	8.86	***<0.00001***	4.31	2.28	8.15	*<0.00001*
Moderate anemia <11 g/dL	557	45	8.07	1.89	1.07	3.35	*0.02*	1.63	0.90	2.95	*0.10*
**Peripheral malaria**											
Positive	97	39	40,2	8.87	5.6	14.05	***<0.00001***	8.52	5.31	13.66	*<0.00001*
Negative	1220	78	6,39	1	-	-	-	1	-	-	-

## Discussion

Five years after implementation of IPT-sp in Côte d’Ivoire, coverage with at least two doses of IPT-sp was 43.3%, although WHO recommends two doses IPTsp for *≥*80% of pregnant women [4]. IPTp coverage rate (≥2 doses) was higher in rural areas than in urban areas (47.4 vs 29.8 p < 0.05).

The lower proportion of use of IPT-sp in urban areas could be due to the women being less health conscious during their pregnancy and the quality of antenatal care services received by them before delivery.

The lowest coverage observed in PMI of Yopougon could be due to the fact that this health center receives a large number of pregnant women and may have been hit more severely by the shortage of SP stock.

In other studies, the coverage of IPT-SP ranged from 12.5% to 58.8% [12-15].

Additional factors can also contribute to low SP coverage including poor accessibility to health facilities and poor quality of antenatal service provided [14,16-18].

Free antenatal care, training health care workers, or providing IPT-SP free of charge and directly observed treatment (DOT), should be implemented in our country to increase the access to antenatal care and IPT-SP use as well as other prophylactic methods such as ITNs.

The overall low coverage occurs despite the finding that among 1317, 1190 (90.4%) women received antenatal care and attended enough visits (≥2) during pregnancy. Higher IPTp coverage has been reported even in settings with lower ANC attendance rates [18,19]. High attendance to ANC does not translate to high IPTp coverage [19,20].

The high proportion of women who attended ANC in an eligible gestation of pregnancy and received their first dose of SP who then made a second ANC visit (90%) demonstrates that women’s ANC attendance was not the primary cause for the ineffectiveness of the IPTp strategy observed. Consistent with other studies, our findings suggest that health provider practices rather than women’s ANC attendance are primarily responsible for the ineffectiveness of the IPTp strategy in this setting [18,21-24].

Stock outs of SP, absence of cups and clean drinking water for taking SP by DOT [17], unclear guidelines [21] or confusion among health workers regarding the guidelines [24] and other related health facility factors [15,25] are factors which hamper delivery of IPTp.

During the study, SP was not always available at pharmacy sites and clean cups and water were also not available to administer the drugs in most of study sites (Bonoua, Yaou, Samo, Yopougon, Pk 18). Women should purchase SP from an external pharmacy and take the drugs at home. Regular availability of SP in the health facility is an important factor in improving the use of IPTp and implementation of the DOT scheme. SP for IPTp in public hospitals in Côte d’Ivoire was supplied by the Ministry of Health. However, periodic supply shortages were frequently reported.

In contrast to IPTp, ITN use during pregnancy was very low (3.6%). This is because these women were not offered an ITN at ANC. ANC is a more effective delivery channel for ITNs and IPTp.

Many studies have reported the prevalence of placental malaria in areas of stable endemic malaria transmission in Africa [1,2]. In a review [26], the median prevalence of placental malaria in all gravidae was 26% (range 5-52%). The relatively low prevalence of placental malaria (9%) and malaria at delivery (7.3%) in our study is therefore probably due to the combination of IPTsp, best malaria case management during pregnancy and that most of our participants were multiparous which conferred a relative protection against malaria during pregnancy.

However, the prevalence of placental malaria observed in our study is almost double that reported by Vanga-Bosson *et al.* in a study conducted at the time of delivery in an area where IPTsp coverage rate (*≥*2 doses) was 49.8% [10].

This difference between the two studies may due to different study sites, ITNs use (3.6% 47.9%) and effective use of IPTp-sp during pregnancy.

A low prevalence of peripheral and placental Plasmodium infection at delivery was also observed in Bangui, Central African Republic [27] and in Thailand [28].

In our study there was no difference in regards to prevalence of peripheral and placental *Plasmodium* infection at delivery between participants having taken SP versus those who had not for IPTp. Resistance to SP and/or IPTp-sp compliance could be the reasons of this similarity. Compliance with the recommendation that IPTp drug should be given under supervision was very low in this study. Only about a third of women who received IPTp took SP in the clinic. Reporting receiving SP or having a record of receiving SP also does not mean the drug was taken. We had no method to verify that SP was taken as reported. Allowing pregnant women to take the IPTp drug unsupervised at the clinic or at home makes compliance uncertain and undermines the essence of IPTp.

Many studies have shown SP to be highly effective at reducing peripheral and placental malaria parasite infection in pregnant women as well as improving pregnancy outcomes [29]. The inconsistent findings on the protective effect of IPTp-SP against maternal malaria and other deleterious pregnancy outcomes have been reported in Tanzania [30], Ghana [31] and Nigeria [32].

LBW infants were born to 18.8% (22/107) of women with placental malaria and 8.5% (103/1097) of women without placental malaria. Women with microscopically detectable placental *P. falciparum* malaria had a higher risk of delivering a LBW baby than those without detectable placental *P. falciparum* corresponding to a relative risk of 2.9, supporting the strong association between *P. falciparum* infection during pregnancy and LBW*.*

Our data indicate that IPTp-sp is not associated with a reduction in LBW. Some studies have however, highlighted an association [13,33].

HIV infection is known to be associated with an increased risk of malaria and LBW, and it may have affected the occurrence of LBW and maternal anemia. HIV may have acted as a confounder in the present study. In the clinical trial sites, HIV screening was routinely undertaken by the National HIV Control Program during the trial, but the HIV status was only determined for 60% of the women. However, the overall prevalence of HIV infection in the present study area was 4% [34]. WHO IPTp policy has been recently updated. The objective is to simplify guidance by recommending the administration of SP to pregnant women at every scheduled antenatal visit [35] on the basis of a recent meta-analysis showing that three or more doses of SP are more effective than two doses [36]. It’s important to evaluate this new policy to see whether this leads to an improvement in IPTp uptake.

The study has several limitations. First, the study findings are limited in terms of overall generalization and impact since the study might have been faced with recall bias. Furthermore, the study was conducted at the health facilities, and this may lead to selection bias as the situation of pregnant women who do not attend the health facility is not known.

Another limitation in this study is the non assessment of the reliability of IPTp use by evaluating the presence of measurable sulfa in maternal plasma during pregnancy.

Despite these limitations, our data provides useful information for the assessment and implementation of IPTp in Côte d’Ivoire and will also inform policy decision.

## Conclusion

IPTp presents greater challenges to deliver through ANC. Urgent efforts are required to improve service delivery of this important intervention.

The plausible interventions to address the gaps and deficiencies include developing a health promotion package to explain the benefits of SP as IPTp agent. In addition health workers should be provided continuing education and training to improve their knowledge of IPTp strategy and DOT scheme. Activities of health workers manning the antenatal clinics should also be supervised. Efficacy of IPTp-sp needs to be evaluated in a controlled trial.
